# Unmet need for contraception among married women in the Kyrgyz Republic using the datasets from the 2006, 2014 and 2018 Multiple Indicator Cluster Survey: a cross-sectional study

**DOI:** 10.1186/s12889-024-18518-6

**Published:** 2024-04-08

**Authors:** Zuura Dolonbaeva, Souphalak Inthaphatha, Bakyt Dzhangaziev, Mederbek Ismailov, Kimihiro Nishino, Nobuyuki Hamajima, Eiko Yamamoto

**Affiliations:** 1 Department of Healthcare Administration, Nagoya University Graduate School of Medicine, Nagoya, Japan; 2 Ministry of Health of the Kyrgyz Republic, Bishkek, Kyrgyz Republic

**Keywords:** Contraception, Family planning, Kyrgyzstan, Kyrgyz Republic, Multiple cluster indicator survey, Unmet need

## Abstract

**Background:**

Since the beginning of the family program in 1998, the proportion of married women who used contraception has fluctuated. An unmet need for contraception among women in Kyrgyzstan drastically increased from 2006 (1.1%) to 2014 (19.1%), and remained unchanged until 2018 (19.0%). This study aims to re-investigate the prevalence of an unmet need for contraception from 2006 to 2018 in a comprehensive manner, and examine the factors associated with an unmet need for contraception among married women over the course of 12 years in the Kyrgyz Republic.

**Methods:**

This is a cross-sectional study using secondary data that derived from the Multiple Indicator Cluster Survey (MICS). The study employed three datasets from the MICS 2006, 2014, and 2018. The study included a total of 9,229 women aged 15–49 who were married and fecund, and whose status of the met/unmet need for contraception could be identified. Logistic regression was employed to estimate the relationship of an unmet need for contraception with independent factors. A *P* value < 0.05 was set as statistically significant.

**Results:**

The prevalence of an unmet need for contraception was 19.9% in 2006, 20.4% in 2014, and 22.5% in 2018. Across 12 years, all reversible-contraceptive methods for women constantly declined. Although intrauterine devices were the prominent contraceptive method of usage among Kyrgyz women, the trend of usage drastically decreased over time. Factors associated with unmet need for contraception included women’s age, area of residence, mother tongue of household head, age of husband, and number of children ever born.

**Conclusion:**

The unmet need for contraception among married Kyrgyz women slightly increased, and the trend of modern contraceptive usage declined from 2006 to 2018, particularly the use of pills, injections, and intra-uterine devices. Comprehensive sexual health education for young people and youth-friendly services should be promoted. An effective and reliable supply chain of contraceptive commodities should be prioritized and strengthened. Regular supportive supervision visits are essential to improve the knowledge and skills of healthcare providers to be able to provide intrauterine device service as a contraceptive choice for Kyrgyz women.

## Introduction

In 2022, it was estimated that approximately 172 million women globally wanted to avoid pregnancies but did not use any methods of contraception [1]. The unmet need for contraception puts women at risk of unintended pregnancies. A global study conducted in 2020 revealed that approximately 121 million women experienced unintended pregnancies every year [2]. While some unintended pregnancies were later welcomed, 61% of unintended pregnancies were unwanted and ended in abortion [2]. Particularly in countries where abortion is banned or illegal, unsafe abortion is often a preferrable choice for unintended pregnant women [2]. Unintended pregnancy is a public health concern because it is associated with maternal morbidity and mortality [3, 4]. In many cultures and countries, it is believed that unintended pregnancy only occurs in unmarried women and is associated with a stigma about becoming pregnant outside marriage. In fact, all fecund and sexually active women and girls can have unintended pregnancies in their lifetime [1].

Encouraging women and girls of reproductive age to practice their rights in reproductive health is one of the global Sustainable Development Goal (SDG) indicators [1, 4]. Contraception is one of the cost-effective investments in reproductive health and rights, which women and girls can exercise their choice on pregnancy [1]. Therefore, the Family Planning 2030 campaign was established to advocate and ensure that women and girls can practice their rights to contraception with appropriate and supportive environments, including legislation, policy, supply chains, and service provisions [5]. Many countries have committed to the global family planning campaign to warrant their progress toward SDGs in 2030, including the Kyrgyz Republic (Kyrgyzstan). To our knowledge, information on contraception in Kyrgyzstan was first reported in 1998 after the country gained its independence [6]. The family planning program was introduced to intervene in the high maternal mortality and infant mortality in the country [6].

After starting the family planning program in 1998, the proportion of married women who used any contraceptive methods fluctuated over the years [7]. The proportion increased from 32.9% (in 1996) to 40.3% (in 1999). However, there was a fluctuation in the contraception consumption rate between the years 2000–2003, then it steadily dropped to 28.6% (in 2012) [7]. While numerous efforts were made to improve the outcome of the family planning program in Kyrgyzstan, an unmet need for contraception among women in Kyrgyzstan drastically increased from 2006 (1.1%) to 2014 (19.1%), and remained unchanged until 2018 (19.0%) [8–10]. As a consequence, it was estimated that approximately 26% of pregnant women in Kyrgyzstan had unintended pregnancy annually [11]. To our knowledge, there have been few comprehensive reports related to contraception in Kyrgyzstan. It is essential to understand what determinants hamper the country’s progression toward its commitments to Family Planning 2030 and SDGs [12]. Therefore, this study aims to re-investigate the prevalence of an unmet need for contraception in a comprehensive manner from 2006 to 2018, and examine the factors associated with an unmet need for contraception among married women over the course of 12 years in Kyrgyzstan.

## Materials and methods

### Multiple indicator cluster survey

The Kyrgyzstan’s Multiple Indicator Cluster Survey (MICS) is a national survey involving women aged 15–49 years from nine regions, including Batkek, Jalal-Abad, Issyk-Kul, Naryn, Osh, Talas, Chui, Bishkek city, and Osh city. It is designed to provide representative information for a large number of indicators, as part of the Global MICS Programme. The surveys were approved by the Ethics Committee of the Ministry of Health of Kyrgyzstan. The MICS in these three survey years were carried out by the National Statistical Committee of Kyrgyz Republic with technical support from the United Nations Children’s Fund (UNICEF). A two-stage sampling technique was employed in this study. In the first stage, the urban–rural level of each region was identified as the main sampling strata. A specified number of clusters was allocated for each area within each stratum. Then, the clusters were drawn systematically with the probability proportional to size at the first sampling stage. After that, lists of households within each cluster were prepared. In the second stage, households were drawn systematically and included in the survey. Sample weight was used for computation and reporting purposes to ensure the accuracy and representativeness of the sample results of all strata [8–10].

### Unmet need for contraception

An unmet need for contraception refers to a fecund and sexually active woman who desires to delay or limit her pregnancy but does not use any methods of contraception [13]. According to the Demographic and Health Surveys Programme, women with an unmet need for contraception refers to the condition in which fecund or sexually active women do not use contraception to fulfil their fertility needs  [13]. An unmet need for contraception is subdivided into two groups for reporting purposes as follows: 1) an unmet need for limiting which refers to women who do not want any more children but do not use contraception; 2) an unmet need for spacing that refers to women who want to delay pregnancy for more than two years but do not use any contraception methods [14]. In contrast, women with a met need for contraception refers to: 1) women who want to become pregnant within two years and do not use any contraception; and 2) women who say they want to delay pregnancy for more than two years or say they do not want any more children and use contraception [14].

### Study design and participants

**Fig. 1:**
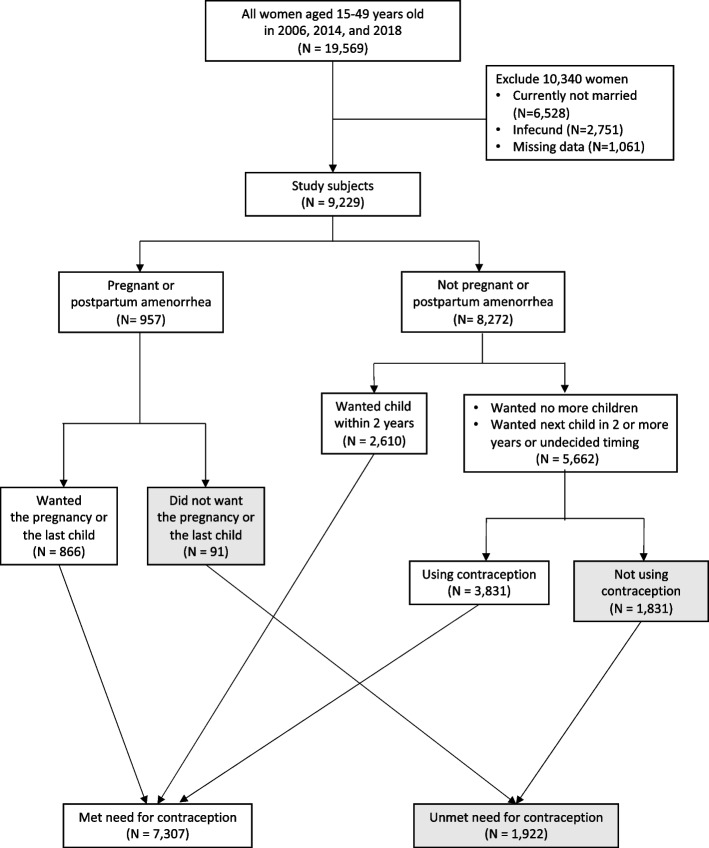
Flowchart of study inclusion and exclusion for unmet need for contraception (Westoff Model, 2012)

This study employed three datasets from MICS 2006, 2014, and 2018, because the same questionnaire related to contraception was used during these times [8–10]. The total number of women enrolled in the MICS surveys were 6,973 in 2006, 6,854 in 2014, and 5,742 in 2018. All women aged 15–49 years old who were currently married and in-union, and fecund were included in this study. Women whose marital status was single, divorced, or widowed, and women who did not use contraception and did not answer on the duration of waiting time for the next child were excluded. Additionally, women who met the criteria of infecund defined by The Demographic and Health Surveys Programme were also excluded from this study [14, 15]. Infecund women were as follows, women who answered ‘cannot get pregnant,’ were postmenopausal, had a hysterectomy, had never menstruated, did not use any contraception but did not have childbirth in the five years preceding the survey, and women who answered ‘last menstruation was before the last pregnancy but last pregnancy was five years prior to the survey.’ Ultimately, a total of 9,229 women were included in this study: 3,287 women in 2006, 3,095 women in 2014, and 2,847 women in 2018 (Fig. 1).

### Outcome variable

The outcome variable was an unmet need for contraception. An unmet need for contraception derived from four questions. For pregnant women or postpartum amenorrhea women who gave birth within two years preceding the survey, a question on desire of the current pregnancy/last infant was asked. If a woman responded that the current pregnancy or the last infant was unwanted or mistimed, they were grouped as having an unmet need for contraception. If the pregnancy/infant was wanted, they were grouped into women with a met need for contraception. For non-pregnant or non-postpartum amenorrheic women, three questions were employed, including the question on current contraception usage, wantedness of future child, and timing of future child if wanted. All non-postpartum amenorrhea women who used contraception at the time of the survey were in the met-need group for contraception. With non-postpartum amenorrhea women who did not use contraception at the time of survey, two probe questions about wantedness of a future child and timing of a future child were employed to determine the status of met/unmet need for contraception. Non-postpartum amenorrhea women who did not decide on the timing for a future child, who wanted a future child in more than two years, and who did not want any more child but did not use any contraception were grouped as ‘unmet need for contraception’. In contrast, women who did not use any contraception but wanted a future child immediately or in less than two years were grouped as having a met need for contraception [14]. The unmet need for contraception was subdivided into two categories, including an unmet need for limiting and an unmet need for spacing. A met-need for contraception was coded as 0, while an unmet need for contraception was coded as 1.

### Operational definition of independent variables

The independent variables included the following: 1) Sociodemographic information (women’s age, education, area of residence, region, mother tongue of household head, and wealth index quintiles). 2) Information regarding reproductive health (age of husband, number of children ever born, and type of contraception use). 3) Attitudes toward domestic violence (DV) of women in this study. Women’s age referred to the age of women at the time of the survey year, it was divided into four groups (15–24, 25–34, 35–44, and 45–49 years old). Women’s education refers to women’s highest education obtained at the time of the survey years, it composed of lower secondary education or lower, high school, and higher education or higher. Area of residence was divided into urban and rural. Region was derived from the question on oblast. Issyk-kul, Naryn, Talas, Chui, and Bishkek C were grouped as North. Batken, Jalal-Abad, Osh, and Osh C were grouped as South. There were three groups for the mother tongue of the household head (Kyrgyz, Russian, and others). Wealth index quintiles referred to standardized household asset scores, it had five categories: poorest, second, middle, fourth, and richest [15]. The age of the husband referred to the age of husband at the time of the survey years, it had four groups (15–24, 25–34, 35–44 and ≥ 45 years old). The number of children ever born was the number of children the women had at the time of the survey year, it was divided into four groups (0, 1, 2 and ≥ 3 children). The types of contraception used among non-pregnant women and non-postpartum amenorrhea women were divided into three groups (modern contraception, traditional contraception, and no methods). Attitudes toward DV had five questions: beating wife is justified if she goes without telling (yes/no), beating wife is justified if she neglects children (yes/no), beating wife is justified if she argues with husband (yes/no), beating wife is justified if she refuses sex (yes/no), and beating wife is justified if she burns food (yes/no). Women who answered ‘no’ to all DV questions were grouped as ‘DV completely unacceptable’. In contrast, women who answered ‘yes’ to all DV questions were grouped as ‘DV completely acceptable’; otherwise, they were grouped as ‘DV somewhat acceptable’.

### Statistical analysis

The analysis was performed using the Statistical Package for the Social Sciences (SPSS) version 29.0 (IBM SPSS Inc.). Sampling weights were included in all analyses of MICS 2006, 2014, and 2018. Logistic regression was employed to estimate the relationship of unmet need for contraception with other factors. A *P* value < 0.05 was set as statistically significant.

### Ethical approval and consent

This study employed secondary data from MICS. All data were anonymous and available online [16]. Approval to access and usage of MICS data was granted by the UNICEF. Since the study used anonymous data, ethical approval for this study was waived.

## Results

### Sociodemographic characteristics of Kyrgyz women

Table 1 shows the sociodemographic characteristics of Kyrgyz women. Most women were aged 25–34 years old (44.3%), had high school education (51.2%), lived in rural areas (63.6%), and the mother tongue of the household head was Kyrgyz (68.8%). The proportion of women aged 15–24 years who were married, fecund, and sexually active slightly decreased from 22.8% (in 2006) to 20.2% (in 2018). In contrast, the proportion of women aged 25–34 years who were married, fecund, and sexually active slightly increased from 43.1% (in 2006) to 45.9% (in 2018). The proportion of women whose household head spoke Russian and other languages gradually decreased over time. On the other hand, Kyrgyz ethnicity increased by 12.5% from 2006 to 2018.

**Table 1:** Sociodemographic characteristics of married Kyrgyz women

	**2006** **(*N* = 3287)** **n (%)**	**2014** **(*N* = 3095)** **n (%)**	**2018** **(*N* = 2847)** **n (%)**	**Total** **(*N* = 9229)** **n (%)**
Women’s age (years old)				
15–24	748 (22.8)	736 (23.8)	576 (20.2)	2060 (22.3)
25–34	1416 (43.1)	1366 (44.1)	1306 (45.9)	4088 (44.3)
35–44	948 (28.8)	836 (27.0)	801 (28.1)	2585 (20.0)
45–49	175 (5.3)	157 (5.1)	164 (5.8)	496 (5.4)
Women’s education				
Lower secondary school or lower	236 (7.2)	318 (10.3)	299 (10.5)	853 (9.2)
High school	2273 (69.2)	1372 (44.3)	1083 (38.1)	4728 (51.2)
Higher education or higher	778 (23.7)	1405 (45.4)	1464 (51.4)	3647 (39.5)
Area of residence				
Urban	1324 (40.3)	1009 (32.6)	1023 (35.9)	3356 (36.4)
Rural	1963 (59.7)	2086 (67.4)	1823 (64.1)	5872 (63.6)
Region				
North	1697 (51.6)	1509 (48.8)	1418 (49.8)	4624 (50.1)
South	1590 (48.4)	1585 (51.2)	1428 (50.2)	4603 (49.9)
Mother tongue of household head				
Kyrgyz	2015 (61.3)	2240 (72.4)	2099 (73.8)	6354 (68.8)
Russian	385 (11.7)	255 (8.2)	159 (5.6)	799 (8.7)
Uzbek	672 (20.4)	475 (15.3)	463 (16.3)	1610 (17.4)
Others	216 (6.6)	125 (4.0)	125 (4.4)	466 (5.0)
Wealth index quintiles				
Poorest	634 (19.3)	614 (19.8)	585 (20.5)	1833 (19.9)
Second	595 (18.1)	627 (20.3)	570 (20.0)	1792 (19.4)
Middle	652 (19.8)	597 (19.3)	560 (19.7)	1809 (19.6)
Fourth	671 (20.4)	610 (19.7)	595 (20.9)	1876 (20.3)
Richest	735 (22.4)	648 (20.9)	537 (18.9)	1920 (20.8)

### Reproductive health and attitudes toward DV

Table 2 shows information regarding reproductive health and attitudes toward DV. Almost half of the women had husbands/partners aged 25–34 years old (44.5%). The majority of Kyrgyz women had three children or more, and the proportion increased from 47.2% in 2006 to 53.6% in 2018. Overall, most women had a met need for contraception (79.2%), but the trend of a met need for contraception gradually declined over time. While the number of women who had an unmet need for limiting slightly decreased, the proportion of women who had an unmet need for spacing increased over time (from 12.0% in 2006 to 16.5% in 2018). The trend in modern contraception usage among non-pregnant women and non-postpartum amenorrhea women declined from 62.1% (in 2006) to 54.7% (in 2018). In contrast, the proportion of women who did not use any contraception methods increased from 34.2% (2006) to 42.4% (2018). Regarding attitudes toward DV, the proportion of women who did not accept any form of DV increased by 14% over the 12 years of the survey.

**Table 2:** Information regarding reproductive health among married Kyrgyz women

	**2006** **(*N* = 3287)** **n (%)**	**2014** **(*N* = 3095)** **n (%)**	**2018** **(*N* = 2847)** **n (%)**	**Total** **(*N* = 9229)** **n (%)**
Age of husband/partner (years old)				
15–24	275 (8.4)	234 (7.6)	158 (5.6)	667 (7.2)
25–34	1430 (43.5)	1401 (45.3)	1271 (44.7)	4102 (44.5)
35–44	1131 (34.4)	1043 (33.7)	976 (34.3)	3150 (34.1)
≥ 45	452 (13.7)	417 (13.5)	440 (15.5)	1309 (14.2)
Number of children ever born				
0	244 (7.4)	263 (8.5)	205 (7.2)	712 (7.7)
1	684 (20.8)	525 (17.0)	407 (14.3)	1616 (17.5)
2	809 (24.6)	793 (25.6)	709 (24.9)	2311 (25.0)
≥ 3	1550 (47.2)	1514 (48.9)	1526 (53.6)	4590 (49.7)
Need for contraception				
Met need	2634 (80.1)	2466 (79.7)	2207 (77.5)	7307 (79.2)
Unmet need for spacing	394 (12.0)	472 (15.3)	469 (16.5)	1335 (14.5)
Unmet need for limiting	259 (7.9)	157 (5.1)	171 (6.0)	587 (6.4)
Type of contraception used among non-pregnant and non-postpartum amenorrhea women (*N* = 8271)^a^				
Modern contraception	1862 (62.1)	1512 (55.9)	1405 (54.7)	4779 (57.8)
Traditional contraception	110 (3.7)	109 (4.0)	73 (2.8)	292 (3.5)
No methods	1027 (34.2)	1083 (40.1)	1090 (42.4)	3200 (38.7)
Beating wife is justified if she goes without telling				
No	2425 (73.8)	2471 (79.8)	2321 (81.5)	7217 (78.2)
Yes	862 (26.2)	624 (20.2)	526 (18.5)	2012 (21.8)
Beating wife is justified if she neglects children				
No	2390 (72.7)	2232 (72.1)	2096 (73.6)	6718 (72.8)
Yes	897 (27.3)	863 (27.9)	750 (26.4)	2510 (27.2)
Beating wife is justified if she argues with husband				
No	2219 (67.5)	2567 (83.0)	2307 (81.0)	7093 (76.9)
Yes	1068 (32.5)	527 (17.0)	540 (19.0)	2135 (23.1)
Beating wife is justified if she refuses sex				
No	2881 (87.6)	2835 (91.6)	2560 (89.9)	8276 (89.7)
Yes	406 (12.4)	260 (8.4)	287 (10.1)	953 (10.3)
Beating wife is justified if she burns food				
No	2839 (86.4)	2859 (92.4)	2659 (93.4)	8357 (90.6)
Yes	448 (13.6)	236 (7.6)	187 (6.6)	871 (9.4)
Attitude toward DV				
DV completely unacceptable	1778 (54.1)	1950 (63.0)	1937 (68.1)	5665 (61.4)
DV somehow acceptable	1371 (41.7)	1073 (34.7)	806 (28.3)	3250 (35.2)
DV completely acceptable	138 (4.2)	71 (2.3)	103 (3.6)	312 (3.4)

*DV* Domestic violence

^a^958 women were pregnant at the time of the surveys and were not asked about contraception methods. There were 288 pregnant women in 2006, 391 pregnant women in 2014, and 278 pregnant women in 2018

### Characteristics of contraception usage

**Fig. 2:**
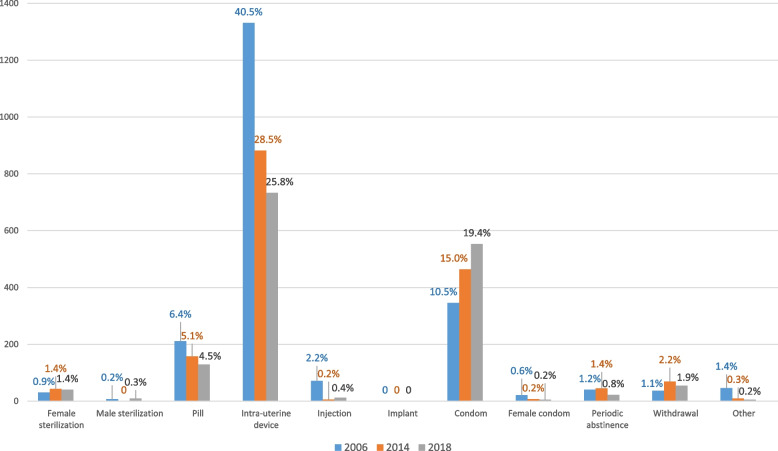
Distribution of contraception usage among Kyrgyz women from 2006, 2014, and 2018

The distribution of contraception usage among Kyrgyz women is shown in Fig. 2. All reversible contraceptive methods constantly declined over time, including pills, injections, and intrauterine device (IUD). None of the women reported using implants in Kyrgyzstan. IUD was the prominent method among Kyrgyz women; however, the trend drastically decreased from 2006 (40.5%) to 2018 (25.8%). While the trends for pills and injection usage also declined, the proportion of male condom use in 2018 was almost double of that in 2006.

### Factors associated with unmet need for contraception

Table 3 shows the multivariate analysis of factors associated with unmet need for contraception in each survey year and across all three survey years. Across three survey years, factors significantly associated with an unmet need for contraception included age of women, area of residence, mother tongue of household head, age of husband, and number of children ever born. Women whose age were 25–34 years, 35–44 years, and 45–49 years were consistently significantly less likely to have unmet need for contraception. After combining the three survey years together, women whom aged 25–34 years old (AOR = 0.60, 95%CI 0.51–0.71, *P* < 0.001), aged 35–44 years old (AOR = 0.36, 95%CI 0.28–0.46, *P* < 0.001), aged 45–49 years old (AOR = 0.22, 95%CI 0.15–0.33, *P* < 0.001) were less likely to have unmet need for contraception compared to women’s age 15–24 years old. While women whose head of household spoke Russian as their mother tongue shows consistent significant association with less unmet need for contraception from 2006 to 2018, women whose head of household spoke Uzbek shows significant association only in 2006 and 2018. When combining all three survey years, women whose head of household spoke Russian (AOR = 0.62, 95%CI 0.49–0.79, *P* < 0.001), and Uzbek ethnic (AOR = 0.70, 95%CI 0.59–0.82, *P* < 0.001) were significantly less likely to have unmet need compared to women whose household head spoke Kyrgyz. Additionally, number of children ever born did not only show consistent significant association with more unmet need for contraception, but the odds ratio also increased drastically in 2014 and 2018 compared to those in 2006. When combining the three survey years together, women who had one child (AOR = 3.87, 95% CI 2.87–5.22, *P* < 0.001), two children (AOR = 4.84, 95% CI 3.57–6.55, *P* < 0.001), and three children or more (AOR = 7.43, 95% CI 5.43–10.17, *P* < 0.001) were more likely to have unmet need for contraception compared to women who had no children.

**Table 3:** Factors associated with unmet need for contraception from MICS 2006, 2014, 2018 and 2006–2018

Variables	MICS 2006	MICS 2014	MICS 2018	MICS 2006–2018
	AOR (95% CI)	AOR (95% CI)	AOR (95% CI)	AOR (95% CI)
Women’s age (years old)				
15–24	1 (reference)	1 (reference)	1 (reference)	1 (reference)
25–34	0.62 (0.47–0.81)^***^	0.59 (0.44–0.78)^***^	0.58 (0.42–0.78)^***^	0.60 (0.51–0.71)^***^
35–44	0.40 (0.27–0.60)^***^	0.26 (0.17–0.40)^***^	0.44 (0.29–0.68)^***^	0.36 (0.28–0.46)^***^
45–49	0.35 (0.18–0.69)^**^	0.16 (0.08–0.33)^***^	0.18 (0.08–0.39)^***^	0.22 (0.15–0.33)^***^
Women’s education				
Lower secondary school or lower	1 (reference)	1 (reference)	1 (reference)	1 (reference)
High school	0.67 (0.48–0.94)^*^	1.13 (0.80–1.60)	0.97 (0.70–1.34)	0.86 (0.71–1.04)
Higher education or higher	0.64 (0.43–0.93)^*^	1.31 (0.91–1.88)	0.85 (0.61–1.20)	0.88 (0.71–1.07)
Area of residence				
Urban	1 (reference)	1 (reference)	1 (reference)	1 (reference)
Rural	0.90 (0.71–1.14)	0.71 (0.55–0.91)^**^	0.82 (0.63–1.07)	0.82 (0.71–0.94)^**^
Region				
North	1 (reference)	1 (reference)	1 (reference)	1 (reference)
South	1.75 (1.39–2.20)^***^	0.89 (0.72–1.11)	0.91 (0.72–1.15)	1.12 (0.99–1.28)
Mother tongue of household head				
Kyrgyz	1 (reference)	1 (reference)	1 (reference)	1 (reference)
Russian	0.65 (0.46–0.92)^*^	0.62 (0.39–0.97)^*^	0.53 (0.30–0.92)^*^	0.62 (0.49–0.79)^***^
Uzbek	0.57 (0.44–0.74)^***^	0.93 (0.69–1.26)	0.72 (0.53–0.98)^*^	0.70 (0.59–0.82)^***^
Others	0.77 (0.53–1.13)	0.98 (0.60–1.62)	0.79 (0.50–1.26)	0.85 (0.67–1.09)
Wealth index quintiles				
Poorest	1 (reference)	1 (reference)	1 (reference)	1 (reference)
Second	1.35 (1.01–1.79)^*^	1.21 (0.92–1.61)	1.18 (0.89–1.57)	1.23 (1.04–1.44)^*^
Middle	1.08 (0.81–1.45)	1.09 (0.81–1.46)	1.02 (0.75–1.38)	1.08 (0.92–1.28)
Fourth	1.21 (0.88–1.67)	0.80 (0.57–1.12)	0.95 (0.68–1.33)	1.00 (0.83–1.20)
Richest	1.30 (0.89–1.88)	0.95 (0.65–1.38)	0.91 (0.60–1.38)	1.05 (0.84–1.31)
Age of husband/partner (years old)				
15–24	1 (reference)	1 (reference)	1 (reference)	1 (reference)
25–34	0.89 (0.63–1.25)	0.78 (0.53–1.15)	0.78 (0.50–1.23)	0.82 (0.66–1.02)
35–44	0.70 (0.46–1.08)	0.60 (0.38–0.97)^*^	0.66 (0.39–1.12)	0.67 (0.51–0.87)^**^
≥ 45	0.74 (0.42–1.29)	0.71 (0.39–1.29)	0.51 (0.27–0.96)^*^	0.66 (0.47–0.92)^*^
Number of children ever born				
0	1 (reference)	1 (reference)	1 (reference)	1 (reference)
1	2.44 (1.63–3.65)^***^	8.58 (4.12–17.89)^***^	5.40 (2.77–10.55)^***^	3.87 (2.87–5.22)^***^
2	2.24 (1.46–3.44)^***^	14.77 (7.10–30.74)^***^	7.19 (3.68–14.02)^***^	4.84 (3.57–6.55)^***^
≥ 3	2.81 (1.80–4.39)^***^	23.45 (11.07–49.69)^***^	12.61 (6.37–24.96)^***^	7.43 (5.43–10.17)^***^
Attitude toward DV				
DV completely unacceptable	1 (reference)	1 (reference)	1 (reference)	1 (reference)
DV somehow acceptable	0.87 (0.71–1.07)	0.96 (0.79–1.17)	0.87 (0.71–1.08)	0.92 (0.82–1.03)
DV completely acceptable	1.29 (0.84–1.98)	1.01 (0.55–1.85)	1.30 (0.79–2.12)	1.28 (0.97–1.68)

*MICS* Multiple cluster indicator survey, *AOR* Adjusted odds ratio, *CI* Confidence interval, *DV* Domestic violence

^*^P<0.05, ^**^P<0.01, ^***^P<0.001

## Discussion

The overall prevalence of an unmet need for contraception slightly increased from 19.9% in 2006 to 22.5% in 2018. While the unmet need for limiting decreased by 1.9% over 12 years, the unmet need for spacing increased 4.5% from 2006 to 2018. The increase in unmet need was consistent with an increased trend of non-contraceptive users across 12 years of surveys. The increase in the number of non-contraceptive users and increase in the prevalence of an unmet need for contraception may be related to the insufficient supply of contraceptive commodities and lack of confidence in the continuity of the supply [17]. Contraceptive commodities in Kyrgyzstan were largely supplied from donations by humanitarian aid, and lack of stock was often reported [17]. Kyrgyz women could only make decisions in relation to the availability of the humanitarian aid options, and the options could be different or unavailable in the next visit [17]. In this study, the proportion of all reversible contraceptive methods for women constantly decreased from 2006–2018, such as pills, IUD, and injection. The decline might have been affected by an insufficient supply of contraception [18]. Kyrgyz women might not choose any contraceptive methods when their preferred contraceptive choices are not available at the visit, thus, the number of non-contraceptive usage increased, and an unmet need for contraception could occur. An effective and reliable supply chain of contraceptive commodities should be prioritized and strengthened to encourage women who are in need of contraception to be able to use modern contraception [19].

IUD is a long-acting reversible form of contraception [20]. It is commonly used worldwide because of its high efficacy and cost-effectiveness [21]. In the Kyrgyz Republic, IUD has been a prominent contraception method among married Kyrgyz women since 1997 [6, 22]. In our study, IUD was still a dominant choice of contraception until 2018, but the trend drastically declined compared to that of 2006. While an inefficient supply chain may affect IUD provision and the number of Kyrgyz women who used IUD decreased, the confidence in the service provision of healthcare providers may also affect the number of IUD users [17]. It was reported that healthcare providers who had no gynecological background were reluctant to provide IUD services to women despite undergoing IUD training, and that IUD services varied from facility to facility depending on the provider’s comfort and confidence level [17]. Regular supportive supervision visits are essential to improve the knowledge and skills of healthcare providers, and to be able to provide IUD service as a contraceptive choice for Kyrgyz women [23].

Kyrgyz women aged 15–24 years old were more likely to have an unmet need for contraception compared to women in other age groups. This result was consistent with that of previous studies in Ethiopia and Ghana, and the likelihood of women having an unmet need for contraception decreased as the age of the women increased [24, 25]. According to the youth assessment in the Kyrgyz Republic in 2010, Kyrgyz youths had limited access to reliable information from media, and a high youth unemployment rate was of socio-political concern to the country [26]. Moreover, younger Kyrgyz women might not have adequate information regarding sexual health education. Access to health services, particularly contraception, might also have been limited [27]. Coupled with the limited domestic employment opportunities for young people, young Kyrgyz women might feel unready to have a child. Therefore, young women were prone to have an unmet need for contraception [26]. The Ministry of Health should promote youth-friendly services for reproductive health, and provide comprehensive sexual health education via offline and online platforms to ensure that young people can access health information from reliable sources. Youth training, such as professional development courses, should be emphasized and strengthened to equip young women with knowledge and skills for employment. Thus, young women should be empowered to be autonomous and financially and mentally prepared for their pregnancy [1].

In our study, women who had children (1, 2 or ≥ 3 children) were more likely to have an unmet need for contraception compared to women who had no children. The result was partially consistent with a study in Zambia, where women with 0 or 1 child were less likely to have an unmet need compared to women with ≥ 4 children, while there were no differences between women with 0 or 1 child and women with 2 or 3 children [28]. This might be because of the difference in variable categorization between our study and that of Zambia. The study in Zambia divided the categories into four subgroups (0–1 child, 2–3 children, 4–5 children and ≥ 6 children), whereas in our study, four subgroups were divided differently (0 child, 1 child, 2 children, and ≥ 3 children) [28]. In this study, the higher the number of children, the higher the likelihood that women had an unmet need for contraception. This indicates that postpartum women might not have adequate access to information and services regarding contraception compared to women with no children. The contraceptive security of the Kyrgyz Republic in 2006 revealed that a healthcare provider shortage in primary healthcare facilities (PHCs) could affect reproductive healthcare services for postpartum women [17]. As the number of healthcare providers was insufficient, contraceptive services for postpartum women might be neglected in PHCs. Human resource allocation and distribution should prioritize PHC to ensure the continuity of contraception services to Kyrgyz postpartum women.

There were a few strengths highlighted in this study. This was the first study on the determinants of unmet need on contraception in Kyrgyzstan using national level data. The results could be generalized to women nationwide. Also, the unmet need for contraception in 2006 shown in this study (19.9%) was drastically higher than that reported by MICS report in 2006 (1.1%) [8]. This might be because this study adopted an updated comprehensive definition of unmet need for contraception developed in 2012. Thus, the prevalence reported in this study could reflect the prevalence in an accurate manner. Despite the strengths, this study has some limitations. Firstly, women with unknown duration of waiting time for the future child and did not use any contraception were excluded from the study, because an unknown duration of the waiting time did not meet the criteria to define the met/unmet need for contraception. The prevalence of an unmet need for contraception may change if these women were included. However, women who did not answer about duration for waiting time for the next child but they used contraception were still included in the study because the met need for contraception could be defined. Secondly, we did not analyze the level of contraceptive knowledge with unmet need for contraception among the samples, because the MICS 2006 did not include contraceptive knowledge in the survey. The questions on contraceptive knowledge were only included in MICS 2014 onwards. Future research including only MICS 2014 and 2018 is recommended, to examine the relationship between awareness on contraception and contraceptive usage. Lastly, as a cross-sectional study, it was difficult to establish the causal-effect relationship between factors and outcomes.

## Conclusion

The prevalence of an unmet need for contraception among married Kyrgyz women slightly increased over 12 years (19.9% in 2006, 20.4% in 2014, and 22.5% in 2018). From 2006 to 2018, the proportion of women who used all reversible contraceptive methods constantly declined, including the use of pills, injections, and IUD. Factors associated with unmet need for contraception included women’s age, area of residence, mother tongue of household head, age of husband, and number of children ever born. Comprehensive sexual health education for young people and youth-friendly services should be promoted. An effective and reliable supply chain of contraceptive commodities should be prioritized and strengthened to be able to provide contraceptive method of choices to the women when they come for the service. Regular supportive supervision visits are essential to improve the knowledge and skills of healthcare providers to be able to provide an IUD service as a contraceptive choice for Kyrgyz women.

## Data Availability

Data can be downloaded from the MICS website repository after user registration and approval for dataset access. Multiple Indicator Cluster Survey datasets from 2006, 2014 and 2018 are available at https://mics.unicef.org/surveys by using the filter for country search > Kyrgyzstan.

## Abbreviations

MICS: Multiple indicator cluster survey
DV: Domestic violence
IUD: Intrauterine device
SDG: Sustainable development goals
UNICEF: United Nations Children’s Fund
PHC: Primary health care
